# Temporal analysis of the autophagic and apoptotic phenotypes in *Leishmania* parasites

**DOI:** 10.15698/mic2018.09.646

**Published:** 2018-08-01

**Authors:** Louise Basmaciyan, Laurence Berry, Julie Gros, Nadine Azas, Magali Casanova

**Affiliations:** 1 UMR PAM A, Valmis team, 2 rue Angélique Ducoudray, BP 37013, 21070 Dijon Cedex, France.; 2 Dynamique des Interactions Membranaires Normales et Pathologiques, CNRS UMR 5235, University of Montpellier, France.; 3 Aix Marseille Univ, IRD, AP-HM, SSA, VITROME, IHU-Méditerranée Infection, Marseille, France.

**Keywords:** Leishmania, apoptosis, autophagy, markers, staurosporine

## Abstract

The leishmaniases are worldwide neglected tropical diseases caused by parasitic protozoa of the *Leishmania* genus. Different stimuli induce *Leishmania* cell death, but the proteins involved remain poorly understood. Furthermore, confusion often appears between cell death and the cell survival process autophagy, whose phenotype is not clearly defined. In this article, we present a comprehensive and temporal analysis of the cellular events occurring during miltefosine-induced cell death and autophagy in *L. major*. We also provide a list of features in order to clearly identify apoptotic cells, autophagic cells and to distinguish both processes. Furthermore, we demonstrate that autophagy is followed by apoptosis in the absence of nutrients. Finally, we show that cells treated with the generic kinase inhibitor staurosporine express apoptotic as well as autophagic markers and therefore cannot be used as an apoptosis inducer in *Leishmania*. These descriptions lead to a better recognition and understanding of apoptosis and autophagy, enabling their targeting in the development of new anti-leishmanial drugs. These researches also make it possible to better understand these processes in general, through the study of an ancestral eukaryote.

## INTRODUCTION

*Leishmania* are flagellated parasitic protozoa responsible for the leishmaniases, a neglected tropical disease that causes between 20,000 and 30,000 deaths per year. Between 700,000 and 1 million people, mostly among the poorest in the world, are at risk of contracting this disease each year in about 97 countries (Global Health Observatory data from the World Health Organization, September 22, 2017). Different stimuli such as the anti-*Leishmania* drugs miltefosine or curcumin induce a form of cell death 1,2,3. After years of debate, regulated cell death in unicellulars has been largely accepted. Indeed, in *Leishmania*, it has been shown that regulated cell death would regulate parasite density within the vector and mammalian host and would modulate host immunity in favor of successful infection 4. However, the cell death pathways involved and the executioner proteins are poorly understood in *Leishmania*. And even if the phenotype of *Leishmania* apoptotic cells has already been described 5, there is still no clear approach to demonstrate *Leishmania* apoptosis.

On the contrary, macroautophagy (hereafter called autophagy) is a cell survival process, notably allowing cells to survive nutrient depletion or growth factor absence 6. More precisely, it is an intracellular catabolic process that sequesters cytosol and organelles within double-membrane-bound vesicles called autophagosomes for delivery to and degradation within lysosomes 7. The amino acids generated are recycled and used for protein synthesis. Autophagy plays important roles in cellular differentiation, tissue remodeling, growth control, size regulation, mitochondrial homeostasis, cellular immunity, adaptation to stress, and unconventional protein secretion 7,8,9,10. *Leishmania* cells initiate autophagy primarily in two situations: in nutrient deprivation conditions and during differentiation (i) from the procyclic promastigote (extracellular and flagellated form) to the metacyclic promastigote form within the vector insect gut and (ii) from the metacyclic promastigote to the intracellular amastigote form of the parasite within the mammalian host 11,12. The association of autophagy and virulence in *Leishmania*, with autophagy-deficient strains presenting low virulence 13, sheds light on the importance of understanding autophagy in this ancestral eukaryote, notably for the discovery of novel targets of new anti-leishmanial drugs. However, if proteins involved in *Leishmania* autophagy have been described 11,13,14,15, the phenotype of autophagic cells remains largely unknown.

Recently, a complex interplay has been described between autophagy and apoptosis in mammalian cells (reviewed in 16). Intuitively, in the majority of cases, apoptosis, the cell death process, and autophagy, the cell survival process, are mutually inhibitory 16. However, some articles suggest that autophagy can precede or even activate apoptosis, by causing the activation of caspases or the depletion of apoptosis endogenous inhibitors 16. Because of the close relationship between the two processes, confusion often occurs between autophagy and apoptosis.

Furthermore, the effects of the generic protein kinase inhibitor staurosporine in *Leishmania *cells are still controversial. While first described as a pro-apoptotic drug in this parasite 17,18 as in all nucleated mammalian cells 19, it does not induce PI-labeling, even after enormous concentrations of staurosporine 17. Besides, a more recent article has shown that staurosporine can induce the expression of apoptotic markers without cell death in *Leishmania* 20. Thus, the consequences of staurosporine treatment still remain unclear.

In this paper, we present a comprehensive and temporal analysis of the cellular events occurring during miltefosine-induced cell death and autophagy in *L. major* in order to better recognize and better distinguish these two processes, highlighting similarities and differences between them. We also confirmed that most of these cellular events occurred during cell death induced by other molecules. Furthermore, we describe the phenotype of staurosporine-treated cells. Last, we studied the link between *Leishmania* cell death and autophagy and we have shown that autophagic cells entered cell death in the absence of nutrients.

## RESULTS

### Growth inhibition and cell morphology during *Leishmania* miltefosine-induced death and autophagy

In order to induce *Leishmania* cell death, we used miltefosine, as indicated in the literature 20,21,22,23,24,25,26. We confirmed its cell death inducer activity by assessing cell membrane disintegrity, the only currently certified technique for quantifying cell death irrespective of the lethal setting 27. Indeed, we observed a significant increase in the percentage of PI (Propidium Iodide)-positive cells 24 h after addition of 40 µM of miltefosine (Fig. S1A). We already demonstrated that increasing the miltefosine concentration or incubation time induces a significant increase in the percentage of PI-positivity 28.

To induce autophagy, we cultivated cells in starvation conditions: without Fetal Calf Serum (FCS) for 24 h or in PBS for 4 h. In order to confirm that autophagy appeared under these conditions, we quantified the percentage of cells containing autophagosomes, as recommended 27. To do so, we transfected *L. major* with a plasmid containing the sequence of the ubiquitin-like protein ATG8 and the sequence of the Green Fluorescent Protein (GFP) at its 5’ end. It has been demonstrated that *Leishmania* cells expressing ATG8 fused with GFP form GFP-labeled puncta corresponding to autophagosomes 11,12. We observed a significant increase in the percentage of autophagosome-containing cells in both starvation conditions compared to the control (Fig. S1B) 11,12.

**Figure 1:**
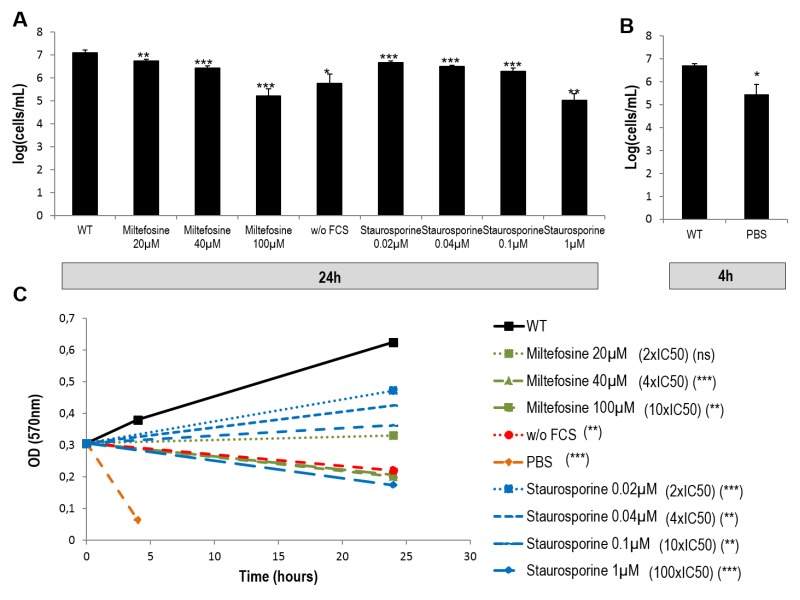
FIGURE 1: Effects on growth and culture density of miltefosine, staurosporine, PBS and of the absence of FCS. **(A-B)** Cell concentration after culture for 24 h in different conditions **(A)** or for 4 h in PBS **(B)**. Mean ± SD from a minimum of three independent experiments. Unpaired t-test between the condition of interest at 24 h and the control condition at 24 h. **(C)** Optical density of cells cultivated in different conditions after addition of MTT. A minimum of three independent experiments were carried out. Unpaired t-test between the condition of interest at 24 h or 4 h and the same condition at 0 h: ns not significant, * p < 0.05, ** p < 0.01, *** p < 0.001.

For the drugs miltefosine and staurosporine, we first calculated the Inhibitory Concentration 50 (IC50) by carrying out an MTT (Methyl Thiazol Tetrazolium) assay. This assay measures the reduction of a tetrazolium salt into formazan by mitochondrial enzymes of living cells. We found an IC50 of 13.2 ± 0.8 µM and 7.2 ± 1.8 nM for miltefosine and staurosporine, respectively. Then, we assessed cell viability in the different cell culture conditions (with miltefosine, without FCS, in PBS and with staurosporine) by counting cells and comparing growth with growth of cells in control conditions. For this, we tested the two drugs at three different concentrations: 2 x IC50, 4 x IC50 and 10 x IC50. As depicted in Figures 1A and B, all conditions induced significant growth inhibition. To clarify the effect of the different culture conditions, we assessed cell vitality by evaluating the consequences on the biosynthetic activity of *L. major* using an MTT assay. Figure 1C shows that miltefosine, at 4 x IC50 and 10 x IC50, not only induced growth inhibition but also a significant decrease in culture density at 24 h compared to 0 h, i.e. efficient killing of the parasites. Therefore, when we attempted to induce *Leishmania* cell death in subsequent experiments, we used miltefosine at 4 x IC50 (40 µM). The autophagic conditions (without FCS for 24 h and PBS for 4 h) also induced significant killing of the parasites when compared to the t0 condition time (Fig. 1C). Regarding staurosporine, this drug, when used at 2 x IC50, 4 x IC50 or even 10 x IC50, reduced cell growth without abrogating it (Fig. 1C). Staurosporine thus had a cytostatic effect on *L. major* cells even at 10 x IC50. When used at a much higher concentration (100 x IC50), staurosporine had a cytotoxic effect, such as the cell death inducing drug miltefosine and the autophagic culture conditions (Fig. 1C). To study whether staurosporine had different consequences depending on the concentration used, further experiments were carried out at 10 x IC50 (0.1 µM) and 100 x IC50 (1 µM).

**Figure 2:**
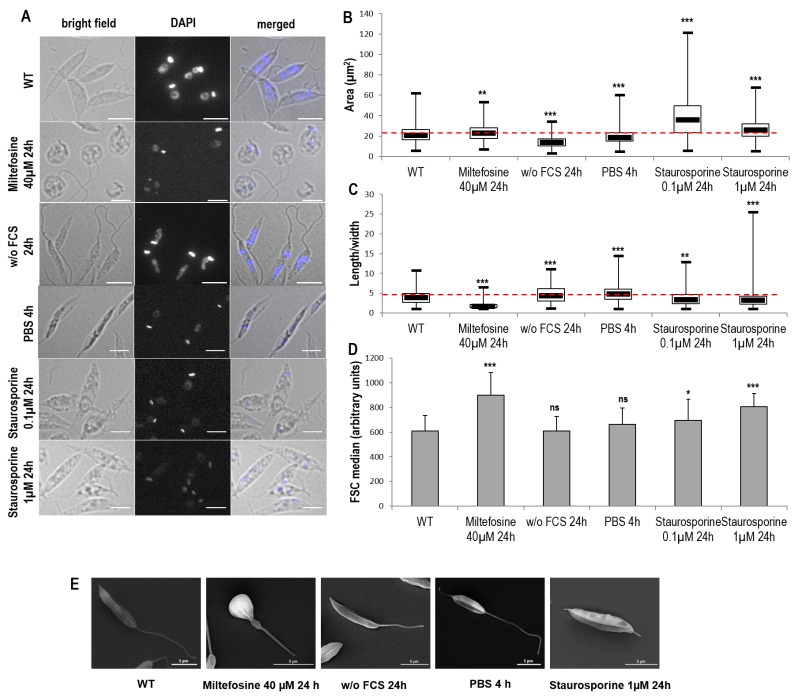
FIGURE 2: Cell morphology during *Leishmania* cell death and autophagy. **(A)** Microscopical observation of cells in different culture conditions: bright field, DAPI and bright field/DAPI merged image (bar = 5µm). **(B-C)** Box plots representing area **(B)** and length-to-width ratio **(C)** of cells cultivated in different conditions. A minimum of 475 cells were counted for each condition, from a minimum of three independent experiments. The thick line inside each box represents the median value; the lower and upper edge of each box indicates the 25^th^ and 75^th^ percentiles, respectively; the lower and upper whiskers (ends of the box arms) represent the minimum and maximum, respectively. **(D)** FSC median calculated by flow cytometry of cells cultivated in different conditions. Mean ± SD from a minimum of thirteen independent experiments. **(E)** Scanning electron microscopy representative images (bar = 5 µm). Unpaired t-test: ns not significant, * p < 0.05, ** p < 0.01, *** p < 0.001.

In a second step, we assessed the effects of cell death and autophagy on the morphology of *L. major* cells. We discovered that miltefosine induced a small but significant increase of cell area accompanied by significant cell rounding up, as shown by fluorescence microscopy and length-to-width ratio decrease (Fig. 2A, B and C). Both autophagic conditions induced a significant cell area decrease accompanied by cell elongation (Fig. 2A, B and C). Concerning staurosporine, it induced a significant increase in cell area, particularly at 0.1 µM, with a small, but significant, cell rounding up (Fig. 2A, B and C). The effects of the different conditions on cell morphology were confirmed by measuring the Forward Scatter (FSC) by flow cytometry. We observed a significant increase in FSC when cells were cultivated with miltefosine and staurosporine but not under autophagic conditions (Fig. 2D), whereas an increase in FSC indicates cell shrinkage 5.

Scanning electron microscopy (SEM) confirmed this morphology, notably a clear cell rounding up in the presence of miltefosine, cell elongation in starvation conditions (with a thin peak in the posterior region when cells were cultivated with PBS) and neither significant rounding up nor elongation for cells treated with 1 µM of staurosporine (Fig. 2E).

### Membrane properties and autophagosome formation during *Leishmania* miltefosine-induced cell death and autophagy

**Figure 3:**
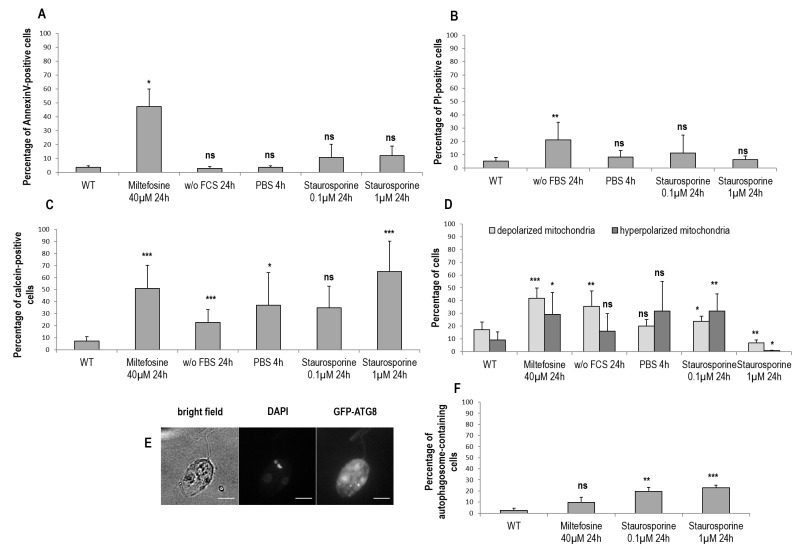
FIGURE 3: Membrane properties and autophagosome formation during *Leishmania* cell death and autophagy. **(A)** Percentage of Annexin V-positive cells in different culture conditions (≥ three independent experiments). **(B)** Percentage of PI-positive cells in different culture conditions (≥ three independent experiments). **(C)** Percentage of calcein-positive cells in different culture conditions (≥ three independent experiments). **(D)** Percentage of cells with a depolarized or hyperpolarized mitochondrion in different culture conditions determined by DIOC_6_ staining (≥ three independent experiments). **(E)** Microscopical observation of cells transfected with GFP-ATG8 and treated for 24 h with 0.1 µM of staurosporine (bar = 5 µm). The GFP-ATG8 puncta are indicative of autophagosomes. **(F)** Percentage of autophagosome-containing cells after treatment for 24 h with miltefosine and with staurosporine or not (WT). A minimum of 722 cells were counted, from three independent experiments. Unpaired t-test: ns not significant, * p < 0.05, ** p < 0.01, *** p < 0.001.

To assess membrane properties during cell death and autophagy, we tested different markers by flow cytometry. In a first time, we quantified the percentage of Annexin V-positive cells. In *Leishmania*, Annexin V is not a cell death marker, owing to the absence of phosphatidylserine that conventionally switches from the inner to the outer leaflet of the plasma membrane during apoptosis 29. However, since Annexin V binds to other membrane lipids, it is indicative of membrane modifications in *Leishmania* 29. We observed that dead cells were significantly Annexin V-positive, contrary to autophagic cells and cells cultivated with staurosporine (Fig. 3A). However, when we investigated PI staining, which is related to membrane integrity, we could observe that not only were dead cells significantly PI-positive, but also cells cultivated for 24 h without FCS (Fig. 3B). On the contrary, cells treated with staurosporine for 24 h were not significantly labeled by PI (Fig. 3B).

We previously demonstrated that calcein is an apoptotic marker in *Leishmania* 28. Therefore, we decided to quantify the percentage of calcein-positive cells in the different conditions studied. We observed that death, as well as autophagy conditions, induced a significant increase in the percentage of calcein-positive cells (Fig. 3C). Concerning staurosporine, while it had no significant effect at 0.1 µM, it induced significant calcein staining at 1 µM (Fig. 3C).

We then quantified the percentage of cells with a depolarized or hyperpolarized mitochondrion, as depicted in supplemental Fig. S2. We observed that PBS for 4 h induced no significant change, while all other conditions induced either a significant mitochondrial depolarization or hyperpolarization (Fig. 3D). We can note that hyperpolarization can be seen in apoptotic cells in addition to depolarization. This has been described as ‘the last attempt by the cells to avoid death’ 5.

To better understand the consequences of staurosporine on *L. major* cells, we evaluated the percentage of cells containing autophagosomes. As presented in Fig. 3E and F, cells cultivated with 0.1 µM or 1 µM of staurosporine significantly contained autophagosomes, as in cells in autophagy, and unlike cells treated with miltefosine.

### DNA degradation during *Leishmania* miltefosine-induced cell death and autophagy

**Figure 4:**
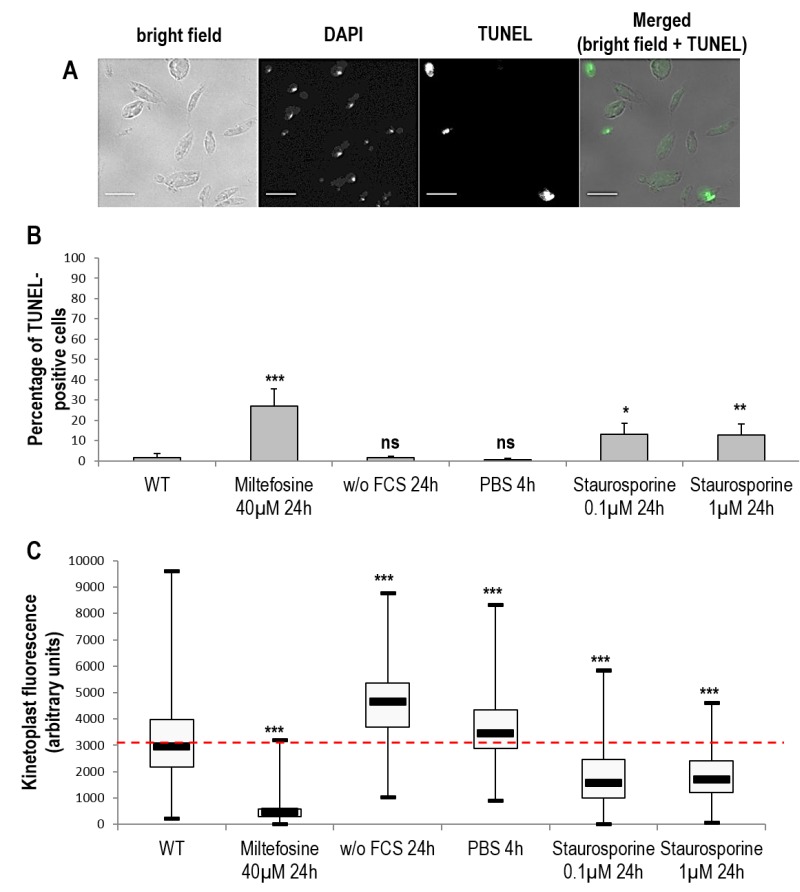
FIGURE 4: DNA degradation during *Leishmania* cell death and autophagy. **(A)** Microscopical observation of cells treated for 24 h with 0.1 µM of staurosporine and stained with DAPI and TUNEL (bar = 5 µm). TUNEL-positive nuclei indicate cells with a degraded nuclear DNA. **(B)** Percentage of TUNEL-positive cells in different cell culture conditions. A minimum of 1800 cells were counted from a minimum of four independent experiments. **(C)** Maximal kinetoplast fluorescence of cells in different culture conditions. A minimum of 200 cells were counted from minimum three independent experiments. Unpaired t-test, * p < 0.05, ** p < 0.01, *** p < 0.001.

We then evaluated the percentage of cells with fragmented DNA. For assessing nuclear DNA fragmentation, we carried out a TUNEL assay and we found that a significant proportion of TUNEL-positive cells appeared when cells were cultivated with miltefosine and also with staurosporine (Fig. 4A and B). In contrast, cells cultivated in starvation conditions presented no nuclear DNA fragmentation (Fig. 4B). *Leishmania* cells are unusual in that they contain a large mitochondrion with a DNA composed of mini- and maxi-circles called the kinetoplast; it is so condensed it is easily observable with DAPI staining. For assessing mitochondrial DNA fragmentation, we measured the maximum fluorescence of the kinetoplast on DAPI fluorescence images. As shown in Fig. 4C, kinetoplast fluorescence was drastically reduced in miltefosine-induced dead cells, indicating high mitochondrial DNA fragmentation. On the other hand, kinetoplast fluorescence was significantly increased in autophagic cells (Fig. 4C). When cultivating cells with staurosporine, kinetoplast fluorescence was reduced, as in dead cells (Fig. 4B).

### Motility during *Leishmania* miltefosine-induced death and autophagy

Motility defects could be clearly observed in autophagic cells, while they were less obvious in miltefosine-treated cells. Video microscopy coupled with live imaging analysis revealed that a large majority of autophagic cells lost their capacity of forward movement: the cells swirled, moved in circles or became motionless, with a beating flagellum (Fig. S3A). During cell death, cell trajectory was not altered, the modifications being insignificant (Fig. S3A). We noticed that, in miltefosine-treated cells, flagellum shortened (Fig. S3B and 2E) and that the region of insertion of the flagellum into the cellular body frequently produced a bulb that was not detected in other conditions (Fig. 2E). A significant reduction in flagellum length was observed for cells cultivated without FCS, while cells cultivated in PBS or with staurosporine showed no flagellum length defects (Fig. S3B).

### Electron microscopy of *Leishmania* cells during miltefosine-induced death and autophagy

**Figure 5:**
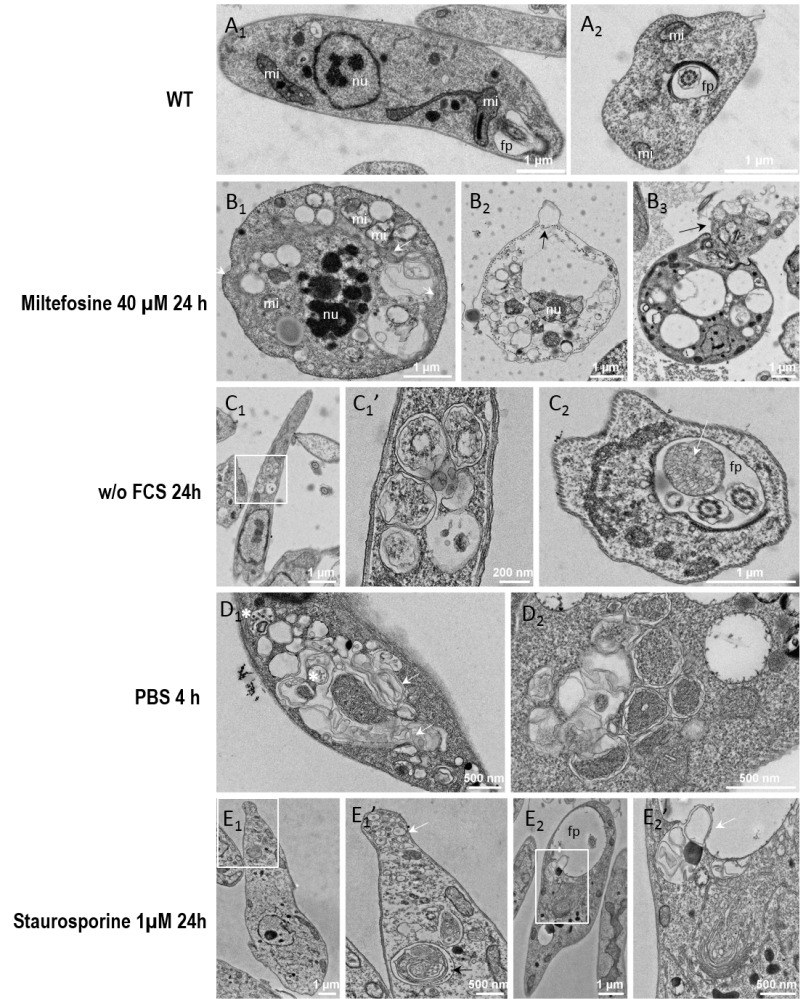
FIGURE 5: Electron micrographs of *Leishmania* cells during cell death and autophagy. Parasites were cultivated for 24 hours in standard conditions **(A_1-2_)**, with 40 µM of miltefosine **(B_1-3_)**, in a medium without FCS **(C_1-2_)**, for 4 h in PBS **(D_1-2_)**, or with 1 µM of staurosporine **(E_1-2_)**. On images **C_1_**, **E_1_** and **E_2_**, the white rectangle indicates a region of interest that was imaged at higher magnification shown on the next image. **(A_1_)** Longitudinal section of a normal parasite, showing the nucleus, 2 sections of the mitochondrion and the flagellar pocket containing a section of the flagellum. Note that the lumen of the flagellar pocket is electrolucent and does not show any cellular material except the flagellum. The cytoplasm does not usually contain accumulation of simple or double membrane vesicles. **(A_2_)** Transversal section through the flagellar pocket of another normal parasite. **(B_1_)** Apoptotic cell showing a shrunk nucleus with patches of highly condensed chromatin (nu) and swollen mitochondrion (mi). The plasma membrane and the subpellicular microtubule network are intact. The cytoplasm is highly vacuolated and shows accumulation of internal membrane multilayers (white arrow). **(B_2_)** Cell in late apoptosis or necrosis. Cytosolic components were essentially released, leaving degraded membranous compartments and a shrunk nucleus. Plasma membrane is making blebs (black arrow). Cortical microtubules are still visible but are not attached any longer to the plasma membrane. **(B_3_)** Apoptotic cell showing abnormal flagellar pocket partially extruding out of the cellular body (black arrow). **(C_1_)** *Leishmania* cells grown for 24 hours in the absence of FCS **(C_1_)** and higher magnification **(C_1_’)**. An accumulation of double membrane vacuoles enclosing cytoplasmic material is observed. **(C_2_)** Cross-section of the flagellar pocket of a cell cultivated without FCS, showing a giant multivesicular body (white arrow). **(D_1_-D_2_)** Parasites incubated in PBS for 4 hours present accumulations of vacuoles containing membrane whorls (white arrows) and multivesicular bodies (white asterisk) **(D_1_)** and double membrane structures enclosing cytoplasm **(D_2_)**. **(E_1_)** Parasites treated for 24 hours with 1 µM of staurosporine contain small vesicles accumulating at the tip of the cell (white arrow). Complex multimembrane structures containing organelles and cytoplasm can also be observed in vacuoles (black arrow). **(E_2_)** The cells present a dilated flagellar pocket. A detail shown in **(E_2_’)**, at higher magnification, shows a cytosolic vacuole containing membrane structures in a process of fusion or fission with the flagellar pocket. nu: nucleus; mi: mitochondrion; fp: flagellar pocket.

To illustrate the morphological changes associated with *Leishmania* death and autophagy, we conducted electron microscopy experiments. Electron micrographs of *Leishmania* cells cultivated for 24 hours with miltefosine showed different stages of cell death. Some cells showed a shrunk nucleus with patches of highly condensed chromatin and a swollen mitochondrion (Fig. 5B1). The cytoplasm was often highly vacuolated and contained an accumulation of internal multilayer membranes (Fig. 5B). Conversely, the plasma membrane and the subpellicular microtubule network were intact. Cells in late apoptosis or necrosis had their cytosolic components essentially released, leaving degraded membranous compartments and a shrunken nucleus (Fig. 5B2). The cortical microtubules were still visible but were no longer attached to the plasma membrane which was occasionally making blebs (Fig. 5B2). Some cells treated with miltefosine had an abnormal flagellar pocket partially extruding out of the cellular body (Fig. 5B3), which corresponds to the bulb observed by SEM (Fig. 2E).

In cells grown for 24 hours in the absence of FCS, an accumulation of double membrane vacuoles enclosing cytoplasmic material was observed which could correspond to early autophagic compartments (Fig. 5C1). Abnormal presence of membrane material was often observed in the flagellar pocket, like the giant multivesicular body represented in Figure 5C2. Multivesicular bodies and double membrane structures enclosing cytoplasm were also observed in parasites incubated in PBS for 4 hours, in addition to an accumulation of vacuoles containing membrane whorls (Fig. 5D1 and D2). The general morphology of cells grown in the absence of serum or in PBS appeared similar, but more marked in PBS, as expected.

Parasites treated for 24 hours with 1 µM of staurosporine frequently contained accumulation of small vesicles as shown in the tip of the cell in Fig. 5E1. Complex multimembrane structures containing organelles and cytoplasm were also observed as depicted in Fig. 5E1. The flagellar pocket appeared dilated and vacuoles containing membranes were often found to be in close apposition, occasionally showing a process of fusion or fission (Fig. 5E2). These observations suggest that staurosporine induced morphological changes that cannot be clearly assigned to autophagy or apoptosis. If abnormal multimembrane compartments were noticed, their aspect was different from the typical double membrane compartments observed under starvation condition.

### Phenotype of *Leishmania* amphotericin B, curcumin, H_2_O_2_ and pentamidine-induced death

To determine whether other molecules inducing *Leishmania* cell death induce the same phenotype as miltefosine, we assessed the consequences of the addition of amphotericin B, curcumin, H_2_O_2_ and pentamidine on* Leishmania* cells. We first carried out a viability assay and as shown in Figure S4A, all molecules induced significant growth inhibition. When assessing cell vitality, we saw that all molecules were cytotoxic: they induced not only growth inhibition but also a significant decrease in culture density at 24 hours compared to 0 hours, i.e. efficient killing of the parasites (Fig. S4B). When monitoring plasma membrane integrity by PI staining, we observed a significant increase in the percentage of PI-positive cells after the addition of all four molecules (Fig. S4C), indicative of loss of plasma membrane integrity.

As shown in Figure S5A, all molecules tested induced a significant increase in the percentage of TUNEL-positive cells. Cell shrinkage was also observed after addition of the molecules, as indicated by the significant increase in the FSC median, with the exception of amphotericin B at 6 µM (Fig. S5B). When evaluating calcein labeling after the addition of the molecules, we observed a significant increase in comparison with cells in control condition (Fig. S5C). The four molecules tested also induced a significant decrease in length/width ratio (Fig. S5D), i.e., cell rounding up, as observed through microscopy (Fig. S5E). In a previous article, we demonstrated that labeling *Leishmania* cells with calcein and PI makes it possible to distinguish early apoptosis from late apoptosis and necrosis 28. When carrying out this double labeling with various concentrations of the different molecules, we consistently observed a switch of the population from calcein-/PI-, indicative of health, to calcein+/PI-, indicative of early apoptosis, and then to PI+, i.e., late apoptosis or necrosis, when we increased the molecule concentration (Fig. S5F-I).

### Autophagy is followed by cell death

**Figure 6:**
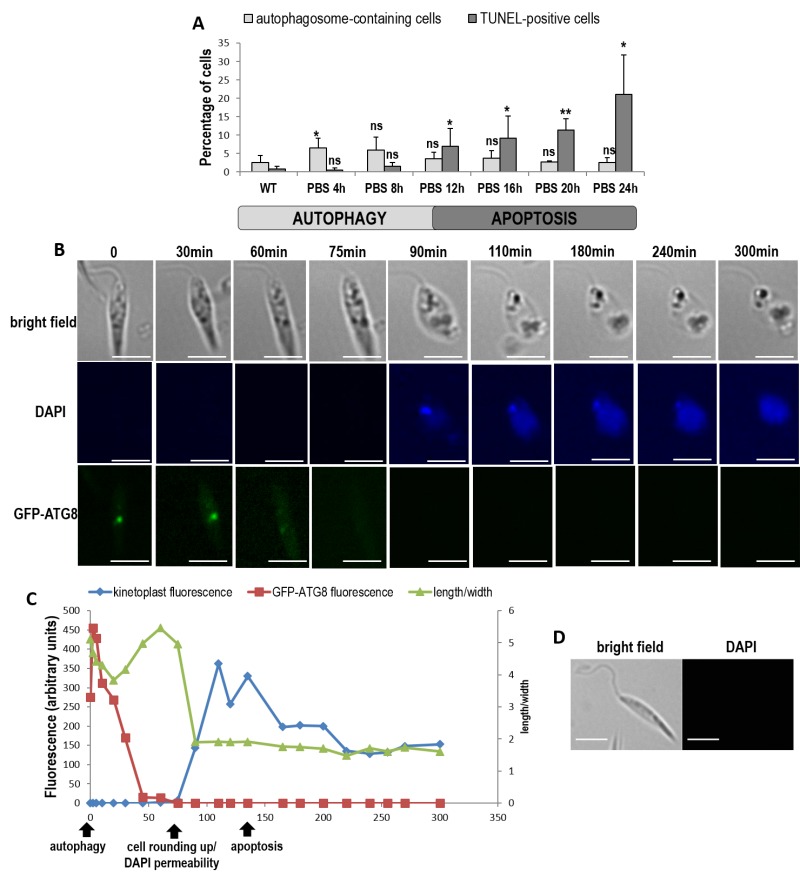
FIGURE 6: When cultivated in PBS, cells first enter autophagy and then die when nutrients are lacking. **(A)** Percentage of autophagosome-containing cells (punctuated ATG8) and TUNEL-positive cells during culture of cells in PBS for 24 h (≥ 1128 cells from a minimum of three independent experiments for the ATG8 assay and from a minimum of five independent experiments for the TUNEL assay). Unpaired t-test: ns not significant, * p < 0.05, ** p < 0.01. **(B)** Real time microscopy of a representative GFP-ATG8 cell cultivated in PBS, showing ATG8 punctuated staining disappearance, nucleus and kinetoplast DAPI staining appearance concomitant with cell rounding up, then kinetoplast fluorescence decrease (bar = 5µm). **(C)** Sequence of events observed in the cell represented in **(B)**, showing the death (characterized by DAPI permeability, cell rounding up and low kinetoplast fluorescence) of an autophagic cell (with GFP-ATG8 puncta). **(D)** Microscopical images of a cell in control conditions (cell cultivated in medium and not in PBS) representative of all the cells observed in the same conditions as in **(B)**, for 300 min (bar = 5 µm).

To better understand the consequences of autophagy, we cultivated cells in PBS for 24 h and assessed autophagy by evaluating the percentage of autophagosome-containing cells (GFP-ATG8 puncta bearing cells) and cell death by evaluating the percentage of TUNEL-positive cells. Figure 6 shows that cultivating cells in PBS induced only autophagy before 12 h, as shown by the appearance of autophagosome-containing cells, while cell death appeared after 12 h of culture in PBS, as demonstrated by the significant appearance of cells with fragmented DNA. This result suggests that cells first entered autophagy when nutrient concentration was decreasing, and died when nutrients were lacking owing to cell consumption.

We then followed specific cells cultivated for 24 h in PBS, by real time microscopy. After 24 h in PBS, about 20% of cells were in apoptosis (Fig. 6A) but since the population is asynchronous, some cells were still in autophagy after 24 h of culture in PBS. In order to follow the autophagic state, we used the strain expressing GFP-ATG8. To evaluate membrane modifications, we put living cells in a mounting liquid (SlowFade Gold antifade mountant) containing DAPI. Because cells were not fixed, DAPI could not stain DNA, except when modifications occurred in the plasma membrane allowing its cell entry. To evaluate the death state, we used the significant decrease of kinetoplast DAPI fluorescence, indicating DNA degradation characterizing cell death, as shown in Fig. 4C. Figure 6B shows a cell representative of all the cells observed. We can see that the cell in autophagy, as shown by the presence of GFP-ATG8 puncta, lost its GFP-ATG8 punctuated staining in PBS. Then, the cell rounded up and, at the same time, became permeable to DAPI. DAPI first intensely stained the kinetoplast before this fluorescence decreased. Yet, cell rounding up, DAPI permeability 5 and kinetoplast DAPI fluorescence decrease are characteristics of cell death in *Leishmania*, as discussed previously. Figure 6C shows the chronology of the different events: autophagosome formation, autophagy exit, then rounding up coinciding with DAPI permeabilization, and kinetoplast degradation, thus cell death. We confirmed that the mounting liquid had no effect on cells, since cells in control conditions remained elongated and DAPI-negative even after 300 min in this liquid (Fig. 6D). This demonstrated that autophagic cells were dying when nutrients were lacking.

## DISCUSSION

**Figure 7:**
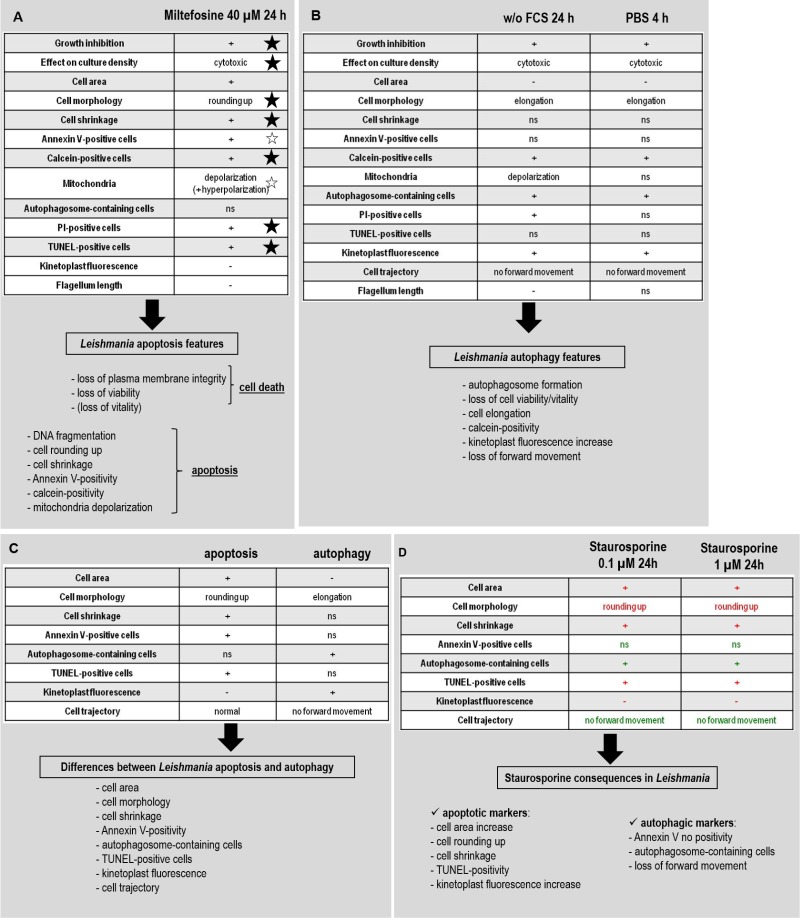
FIGURE 7: Proposed procedure to identify *Leishmania* apoptotic cells, autophagic cells and to clearly distinguish apoptosis and autophagy. **(A)***Leishmania* miltefosine-induced apoptosis markers. A star indicates the apoptotic markers that have been found not only in miltefosine-induced apoptosis but also after induction of apoptosis by other molecules: a solid star when demonstrated in this article and an empty star when shown in the literature (see discussion for details). The proposed approach to demonstrate *Leishmania* apoptosis is also summarized. **(B)** *Leishmania* autophagy markers. The list of features observed in deprivation conditions (without FCS for 24 h and in PBS for 4 h) as well as the proposed approach to demonstrate *Leishmania* autophagy are summarized. **(C)** Features to distinguish apoptosis and autophagy in* Leishmania*. The characteristics that are different during *Leishmania* apoptosis and autophagy and so that allow distinguishing the two processes are enumerated. **(D)** Features induced by staurosporine in *Leishmania*. Staurosporine induces apoptotic (in red) as well as autophagic (in green) features in *Leishmania*.

*Leishmania* are parasitic protozoa that can undergo a form of cell death when subjected to stimuli such as miltefosine. As recommended for cell death characterization in yeast 27, we evaluated the occurrence of cell death after the addition of miltefosine by monitoring the loss of plasma membrane integrity (PI staining), the loss of cell viability (cell growth) and the loss of cell vitality (MTT assay). Secondly, we examined morphological and biochemical features to characterize this form of cell death. We notably observed cell rounding up, DNA fragmentation, plasma membrane modifications with maintenance of its integrity and mitochondrial membrane modifications (Fig. 7A). Since the Nomenclature Committee on Cell Death indicates that the term ‘apoptosis’ describes a specific morphological aspect of cell death and should therefore apply to cell death events that occur while manifesting several apoptotic morphological features 30, we can call this form of miltefosine-induced cell death ‘apoptosis’ in *Leishmania*. For now, the executioner proteins and the metabolic pathways involved are largely unknown, preventing us from assessing the regulatory network as recommended in yeast 27. As a result, some authors prefer talking about incidental cell death rather than regulated cell death for this unicellular parasite 31.

Figure 7A summarizes the characteristics of miltefosine-treated cells. When studying different molecules described as apoptosis inducers in *Leishmania* (amphotericin B, curcumin, H_2_O_2_ and pentamidine) 2,32,33,34, we showed that several features listed in this figure were common to these molecules and miltefosine: growth inhibition, cytotoxicity, cell rounding up, cell shrinkage, calcein-positivity, PI-positivity and TUNEL-positivity. This led us to define not only miltefosine-induced apoptotic markers but *Leishmania* apoptotic markers in general. These common features are marked by a solid star in Figure 7A. Furthermore, Annexin V-positivity has been already described for other apoptotic drugs, notably curcumin 32 and pentamidine 2. Mitochondrion depolarization has also been described after the addition of different molecules described as apoptotic in *Leishmania*, for example H_2_O_2_ 35,36 or amphotericin B 33. As a consequence, as in yeast 27, to demonstrate *Leishmania* apoptosis, we propose to sequentially evaluate: (i) the occurrence of cell death by carrying out a viability assay (growth curve) and monitoring the loss of plasma membrane integrity (PI staining); (ii) the type of cell death by showing the presence of apoptotic markers among DNA fragmentation, cell rounding up, cell shrinkage, Annexin V-positivity, calcein-positivity or mitochondrial depolarization. This procedure is summarized in Figure 7A.

Autophagy is a survival process that generates metabolic substrates that meet the bio-energetic needs of cells, thereby allowing for adaptive protein synthesis, and thus cell survival in nutrient stress conditions 37. *Leishmania* autophagy has been clearly demonstrated during starvation or cell differentiation 11,12. However, the phenotype of autophagic parasites has been poorly described. In this article, we have characterized autophagy in the protozoan

parasite *Leishmania*, in comparison with apoptosis, in order to better understand autophagy and to avoid confusion between both processes. To induce autophagy, we cultivated cells without FCS for 24 h or in PBS for 4 h. Figure 7B summarizes the results we obtained. Based on our results, in order to demonstrate the cell survival process autophagy, we propose to show the presence of autophagic features shared by both conditions tested in this study, among autophagosome formation, loss of cell viability or vitality, cell elongation, calcein positivity, kinetoplast fluorescence increase or loss of forward movement. Despite some similarities (growth inhibition, cytotoxicity and calcein labeling), the phenotype of autophagic and apoptotic cells was clearly distinct. Indeed, while autophagic cells became thinner with a reduction of cell area, apoptosis was characterized by cell rounding up, area increase and cell shrinkage. Unlike autophagic cells, apoptotic cells were Annexin V-positive. In addition, autophagic cells contained autophagosomes, while apoptotic cells did not. The mitochondrial and nuclear DNA of autophagic cells remained intact, while it degraded in apoptotic cells. Finally, autophagic cells were rapidly gyrating or became immobile, while apoptotic cells did not show obvious motility defects. As a consequence, these eight features (that concern cell morphology, Annexin V positivity, DNA fragmentation, autophagosome formation and motility) allowed us distinguishing apoptosis from autophagy, as summarized in Figure 7C. It can be noted that calcein remains a good apoptosis marker, particularly to distinguish early from late apoptosis as previously mentioned 28. However, it cannot be used to distinguish apoptosis from autophagy in *Leishmania* since it also labels autophagic cells. We can also note that the fact that cells cultivated without FCS are PI-positive is really surprising, since PI-labeling is usually associated with cell death. However, when we have a detailed look, we can see that a significant proportion of cells cultivated without FCS were in necrosis (9.6% of calcein-negative/PI-positive cells, while it was 3.8% in cells cultivated in control conditions; pvalue=0.008; data not shown), which explains the PI-positivity.

We showed that under stimuli such as nutrient concentration decrease, cells entered autophagy. But if nutrient stress exceeded a critical threshold, for instance when cells were cultivated for several hours in PBS, cells in autophagy entered apoptosis. This suggests the existence of proteins linking both processes. The close relationship between apoptosis and autophagy has already been described in mammalian cells, as reviewed in Mariño *et al.* 16, being assigned to the dual action of caspases 16 or of proteins of the Bcl-2 family, in apoptosis and autophagy 38. In the ancestral eukaryote *Leishmania*, this crosstalk between apoptosis and autophagy may be mediated by the metacaspase, which plays a role similar to caspases, absent in this parasite, in particular since this enzyme has been described as involved in apoptosis as well as in autophagy in *Leishmania* 39. We can also wonder whether the recently identified Bcl-2 family protein Li-BH3AQP is also involved 40,41.

The generic kinase inhibitor staurosporine is described as an apoptosis inducer in higher eukaryotes 42, as well as in *Leishmania *17,43. However, we here demonstrated that cells treated with staurosporine expressed apoptotic as well as autophagic markers (Fig. 7D). Indeed, staurosporine induced a significant increase in cell area, cell rounding up, cell shrinkage and nuclear and mitochondrial DNA fragmentation, as in apoptotic cells. On the other hand, staurosporine induced formation of autophagosomes, mobility defects with the absence of forward movement and no Annexin V labeling, as in autophagic conditions. It is worth mentioning that these consequences were observed whatever the staurosporine concentration: 10 x IC50 or 100 x IC50. These results confirm the phenotype of staurosporine-treated cells recently described: mitochondrial membrane modification, cell elongation, loss of mobility and absence of cell death 20. We can hypothesize that staurosporine induces *Leishmania* autophagy which is followed, like in PBS as we showed in this article, by apoptosis, explaining why autophagic as well as apoptotic markers are found in an asynchronous culture of *Leishmania* cells. In any case, due to its highly complex response that does not only involve apoptosis, staurosporine cannot be used as an apoptosis-inducer in *Leishmania*.

## Conclusion

This article describes the phenotype of *Leishmania* cells in apoptosis and in autophagy. It will help identifying and discriminating both processes, using a list of specific markers. It also demonstrates that autophagic cells enter apoptosis in the absence of nutrients. Finally, it confirms the pleiotropic consequences of the protein kinase inhibitor staurosporine, demonstrating that it cannot be used as neither a pure autophagy nor apoptosis-inducer in *Leishmania*.

Autophagy, as well as apoptosis, constitutes a target of choice for the identification of new anti-leishmanial drugs. Indeed, it is closely linked to apoptosis, its importance in virulence has been demonstrated 13 and its pathway is highly specific to *Leishmania* 15. The study of original models such as ancestral eukaryotes may also provide a better understanding of the biology of these processes, facilitating the development of autophagy-based treatments for human diseases such as cancer, in which changes in autophagy activity are often involved (reviewed in 44).

## MATERIALS AND METHODS

### Parasites

*L. major* ‘Friedlin’ promastigotes (MHOM/IL/81/Friedlin) were grown in Schneider’s *Drosophila* medium (Life Technologies, Saint-Aubin, France) supplemented with 100 U/mL penicillin, 100 µg/mL streptomycin, 2 mM glutamine and 20% FCS (Life Technologies, Saint-Aubin, France) at 26°C.

### Drug treatment

Logarithmic *L. major* cells were incubated with amphotericin B (Sigma-Aldrich, Saint-Louis, MO, USA), curcumin (Sigma-Aldrich), H_2_O_2_ (Sigma-Aldrich), miltefosine (Santa Cruz Biotechnology, Dallas, TX, USA), pentamidine (Sigma-Aldrich) or staurosporine (Sigma-Aldrich) for 24 h at 5 x 10^6 ^cells/mL for the IC50 and growth inhibition experiments, or at 10^7 ^cells/mL for all other experiments.

### Induction of autophagy 

For nutrient deprivation, logarithmic *L. major* cells were harvested by centrifugation at 600 *g* for 10 min, washed once with sterile PBS and incubated at 10^7 ^cells/mL, either in a serum-deprived medium or in PBS.

### IC50

To calculate the drug concentration inhibiting 50% of cell growth (IC50), an MTT assay was carried out. For this, cells were incubated in triplicate at a density of 10^6^ cells/mL in a 96-well plate with various concentrations of the drugs dissolved either in H_2_O (for H_2_O_2_, miltefosine and pentamidine), or in ethanol (for curcumin), or in DMSO (for amphotericin B and staurosporine). Appropriate controls (DMSO or H_2_O alone) were added. After 72 h of incubation at 26°C, 20 µL of MTT (Sigma-Aldrich, Saint-Louis, MO, USA) at 5 mg/mL in PBS was added. After incubation at 26°C for 4 h, 100 µL of a blocking solution was added (10% m/v Sodium Dodecyl Sulfate and 50% v/v isopropanol) and optical density was measured on a spectrophotometer (ELX808 Ultra Microplate Reader, Bio-Tek Instruments, France) at 570 nm.

### Growth inhibition

For evaluating the cytotoxic or cytostatic effect of the different conditions tested, cells were incubated at 5 x 10^6^ cells/mL in different conditions (amphotericin B, curcumin, H_2_O_2_, miltefosine, pentamidine or staurosporine for 24 h, without FCS for 24 h, with PBS for 4 h). Cells were then counted with a Thoma counting chamber and 100 µL from each condition was placed on a 96-well plate in triplicate before adding 20 µL of MTT at 5 mg/mL in PBS. After incubation at 26°C for 4 h, 100 µL of a blocking solution was added (10% m/v Sodium Dodecyl Sulfate and 50% v/v isopropanol). Optical density was measured on a spectrophotometer (ELX808 Ultra Microplate Reader, Bio-Tek Instruments, France) at 570 nm.

### Cell morphology, flagellum length and kinetoplast fluorescence

Harvested cells were fixed in 4% paraformaldehyde, washed in PBS and directly loaded on fluorescence slides before mounting with SlowFade Gold antifade mountant with DAPI (Life Technologies, Saint-Aubin, France). Images were captured using a BX51 fluorescence microscope (Olympus, France) and the fluorescence imaging system Cell^A^ (Olympus, France). The software ImageJ 1.48s was then used for evaluation of cell morphology (area, major and minor axes of the fitted ellipse of each cell), for flagellum length and for kinetoplast fluorescence (max gray value of the kinetoplast).

### TUNEL

For the TUNEL assay, cells were fixed with paraformaldehyde 4%, laid on an immunoslide and permeabilized with a 0.1% triton X-100 and 0.1% sodium citrate solution for 2 min at 4°C. After three washes in PBS, the enzyme from the *in situ* cell death detection kit, fluorescein (Roche, Meyla, France) was added, diluted 1/10 in the kit buffer. The cells were washed five times in PBS before air drying and addition of SlowFade Gold antifade mountant with DAPI. Cells were observed with a BX51 fluorescence microscope. Bright field and fluorescence images were acquired using the fluorescence imaging system Cell^A^.

### Annexin V

For Annexin V labeling, cells were washed once with PBS and resuspended in 50 µL Binding Buffer (BD Biosciences, France) in which 2.5 µL of AnnexinV BV421 (BD Biosciences, France) and 2.5 µL of PI (Sigma-Aldrich, Saint-Louis, MO, USA) at 0.5 mg/mL were added. Cells were incubated 15 min at room temperature in the dark before adding 200 µL of Binding Buffer. The cells were analyzed by flow cytometry using 488 nm excitation and measuring violet fluorescence emission for Annexin V (450/50 bandpass) and red fluorescence emission for PI (610/20 bandpass) on the BD LSRFortessa™ cell analyzer (BD Biosciences, France). Data were exported and analyzed with Flowjo software for evaluation of the percentage of Annexin V-positive cells and of the FSC median.

### Calcein and PI labeling

Cells were washed once in PBS and resuspended in 1 mL of PBS with 2 µL of calcein (LIVE/DEAD® Viability/Cytotoxicity Kit for mammalian cells, Molecular Probes, OR, USA) diluted 1/80 in DMSO and 4 µL PI at 0.5 mg/mL. The mixed sample was then incubated for 15-20 minutes at room temperature, protected from light. The cells were analyzed by flow cytometry using 488 nm excitation and measuring green fluorescence emission for calcein (530/30 bandpass) and red fluorescence emission for PI (610/20 bandpass) on the BD LSRFortessa™ cell analyzer. Data were exported and analyzed with Flowjo software for evaluation of the percentage of calcein- and PI-positive cells and of the FSC median.

### Autophagosome-containing cells

For the expression of *L. major* cells expressing the autophagosome marker ATG8 fused in N-terminal to GFP, cells were transfected as previously described 39 with the pGL1078 GFP-ATG8 vector constructed by J. Mottram (University of York, UK) and kindly provided by G. van Zandbergen (Paul Ehrlich Institute, Germany). For intracellular localization of ATG8, harvested cells were fixed in 4% paraformaldehyde (4°C, 30 min), washed in PBS and air-dried on microscope fluorescence slides before mounting with SlowFade Gold antifade mountant with DAPI. Observations were done using a BX51 fluorescence microscope and images acquired using the fluorescence imaging system Cell^A^.

### DiOC_6_ fluorescence

For monitoring the mitochondrial membrane potential, cells were washed once in PBS before addition of 60 nM DiOC_6_ (3,3’-dihexyloxacarbocyanine iodide) (Sigma-Aldrich, Saint-Louis, MO, USA). Cells were then incubated for 30 min at 26°C, washed in PBS and resuspended in PBS. DiOC_6_ fluorescence was measured by flow cytometry using 488 nm excitation and measuring green fluorescence emission on the BD LSRFortessa™ cell analyzer. Data were exported and analyzed with Flowjo software for evaluation of the percentage of cells with a depolarized or hyperpolarized mitochondrion and of the FSC median.

### Cell motility

For analysis of cell trajectory and speed, live cells were loaded on slides and a video was recorded for 20 s with Cell^A^. Speed was measured using ImageJ 1.48s.

### Scanning Electron Microscopy

*Leishmania* cells were layered on poly-lysin-coated coverslips, deshydrated in graded series of ethanol followed by critical point drying (CDP-030, BAL-TEC). Coverslips were mounted on specimen stubs, coated with 9 nm of platinum (SCD 050 sputter/coater BAL-TEC) and observed with FEI Quanta FEG200 microscope. All chemicals were from Electron Microscopy Sciences (Hatfield, PA, USA), solvents were from Sigma-Aldrich (Saint-Louis, MO, USA). Images were treated with Fiji and the figures were mounted using the FigureJ plugin.

### Electron microscopy

Sample were fixed in 2.5% glutaraldehyde in 0,1 M phosphate buffer at room temperature, post-fixed for 1 h in 1% osmium tetroxide in the same buffer. Sample were then washed in water, and stained overnight in 2% uranyl acetate in water, deshydrated in graded series of ethanol and propylene oxide and embedded in Epoxy resin (Embed 812). Thin sections were obtained with a Leica Ultracut ultramicrotome. Grids were contrasted with 2% uranyl acetate and lead citrate, and observed with a Jeol 1200 EXII. All chemicals were from Electron Microscopy Sciences (Hatfield, PA, USA), solvents were from Sigma-Aldrich (Saint-Louis, MO, USA). Images were treated with Fiji and the figures were mounted using the FigureJ plugin.

### Real-time microscopy

Cells expressing ATG8-GFP and cultivated for 24 h in PBS were loaded on fluorescence slides and the slides were mounted with SlowFade Gold antifade mountant with DAPI. Images were captured at intervals with the fluorescence microscope and the images were analyzed using ImageJ 1.48s.

### Statistical analysis

The results are presented as mean ± SD (Standard Deviation). For statistical analysis, unpaired t-tests were done (BioStaTGV or Excel). Results were considered statistically significant when p < 0.05. For significant differences, * means p < 0.05, ** p < 0.01 and *** p < 0.001.

## SUPPLEMENTAL MATERIAL

Click here for supplemental data file.

All supplemental data for this article are also available online at http://microbialcell.com/researcharticles/temporal-analysis-of-the-autophagic-and-apoptotic-phenotypes-in-leishmania-parasites.
