# Supplementary material for: Temporal analysis of the autophagic and apoptotic phenotypes in Leishmania parasites
Source: Microb Cell. 2018 Aug 1;5(9):404–17. doi: 10.15698/mic2018.09.646 (PMC6167523; doi:10.15698/mic2018.09.646)
Supplement: Supplementary file 1 [file mic-05-404-s01.pdf]

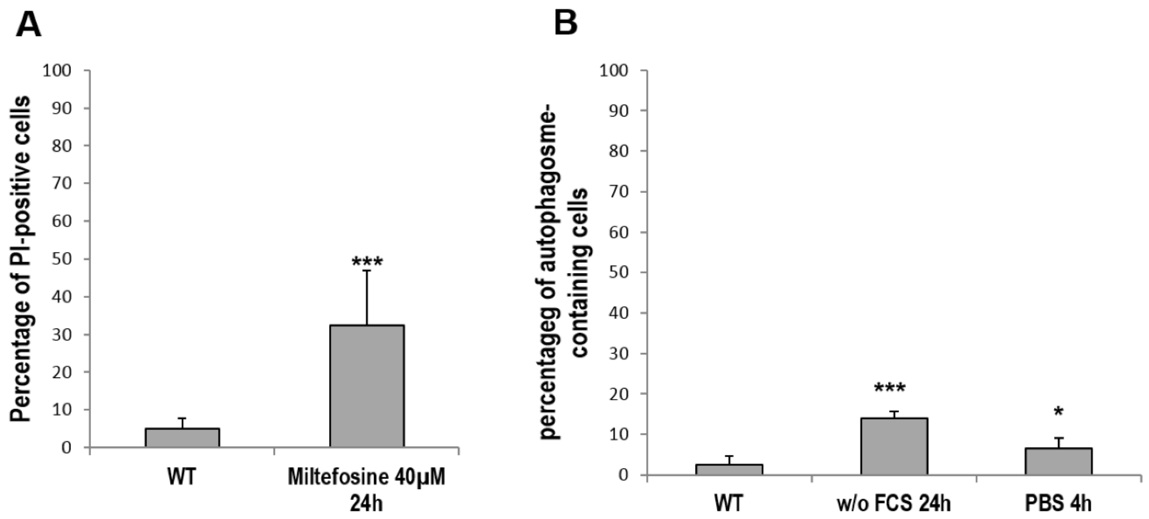

FIGURE S1: Miltefosine induces *Leishmania* cell death while culture in starvation conditions (without FCS or in PBS) induces autophagy. (A) Percentage of cells that have lost their plasma membrane integrity (PI-positive cells) in control conditions and after addition of 40µM of miltefosine for 24 h. A minimum of 9 independent experiments were carried out. **(B)** Percentage of autophagosome-containing cells in control conditions and in different starvation conditions (without FCS for 24 h or in PBS for 4 h). A minimum of 1085 cells were counted from four independent experiments. Unpaired t-test: * $p < 0.05$, *** $p < 0.001$.

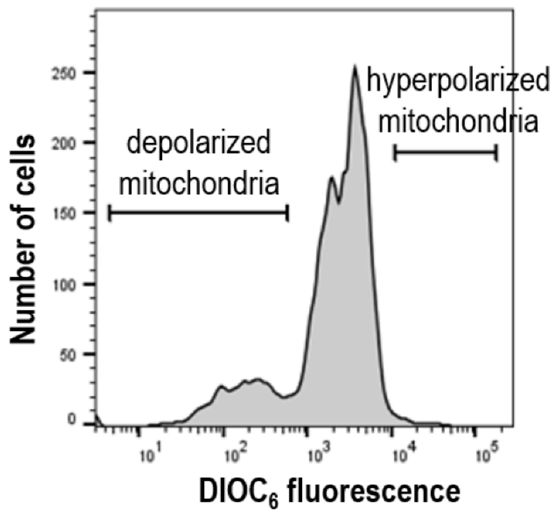

FIGURE S2: Raw histogram showing how the percentage of cells with depolarized and hyperpolarized mitochondrion was quantified by flow cytometry.

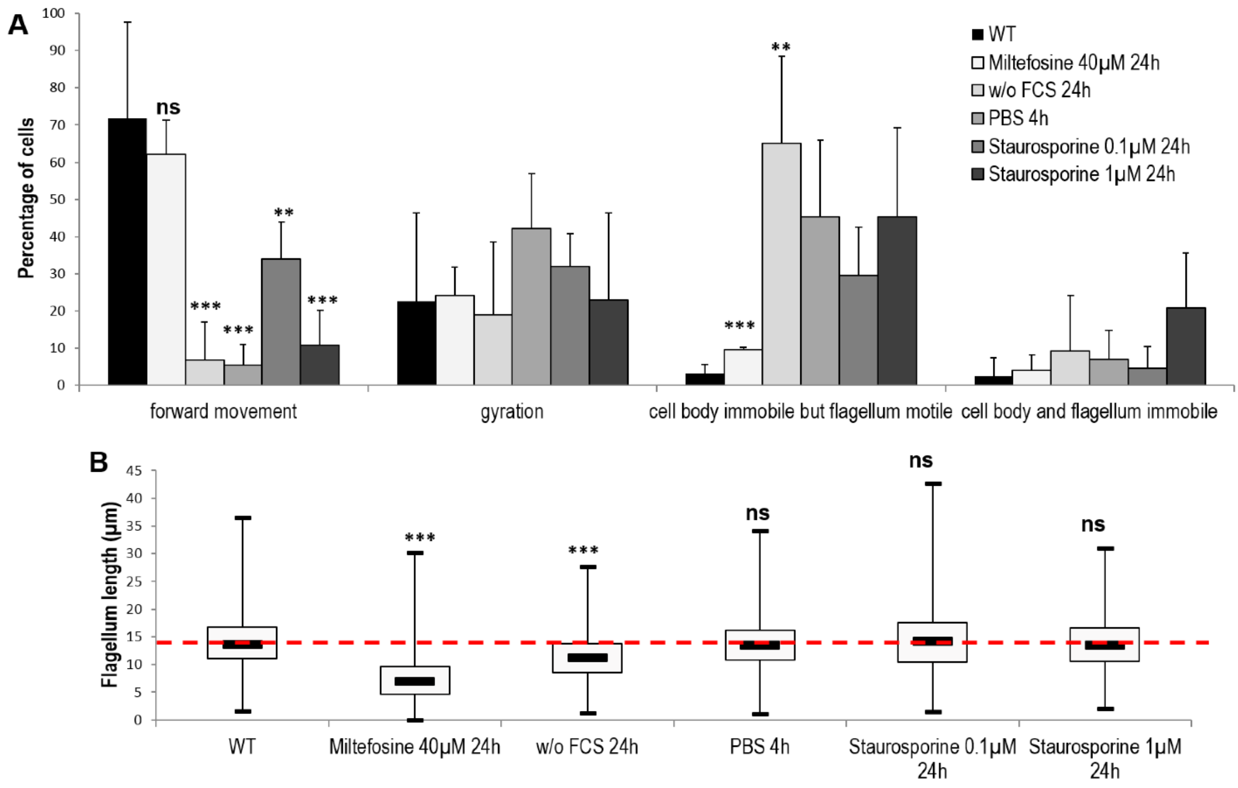

FIGURE S3: Mobility during *Leishmania* death and autophagy. (A) Trajectory of cells in different culture conditions. A minimum of 69 cells were counted for each condition from a minimum of three independent experiments. **(B)** Box plots representing flagellum length of cells. A minimum of 320 cells were counted for each condition from a minimum of three independent experiments. Unpaired t-test: ns not significant, **: $p < 0.01$, ***: $p < 0.001$.

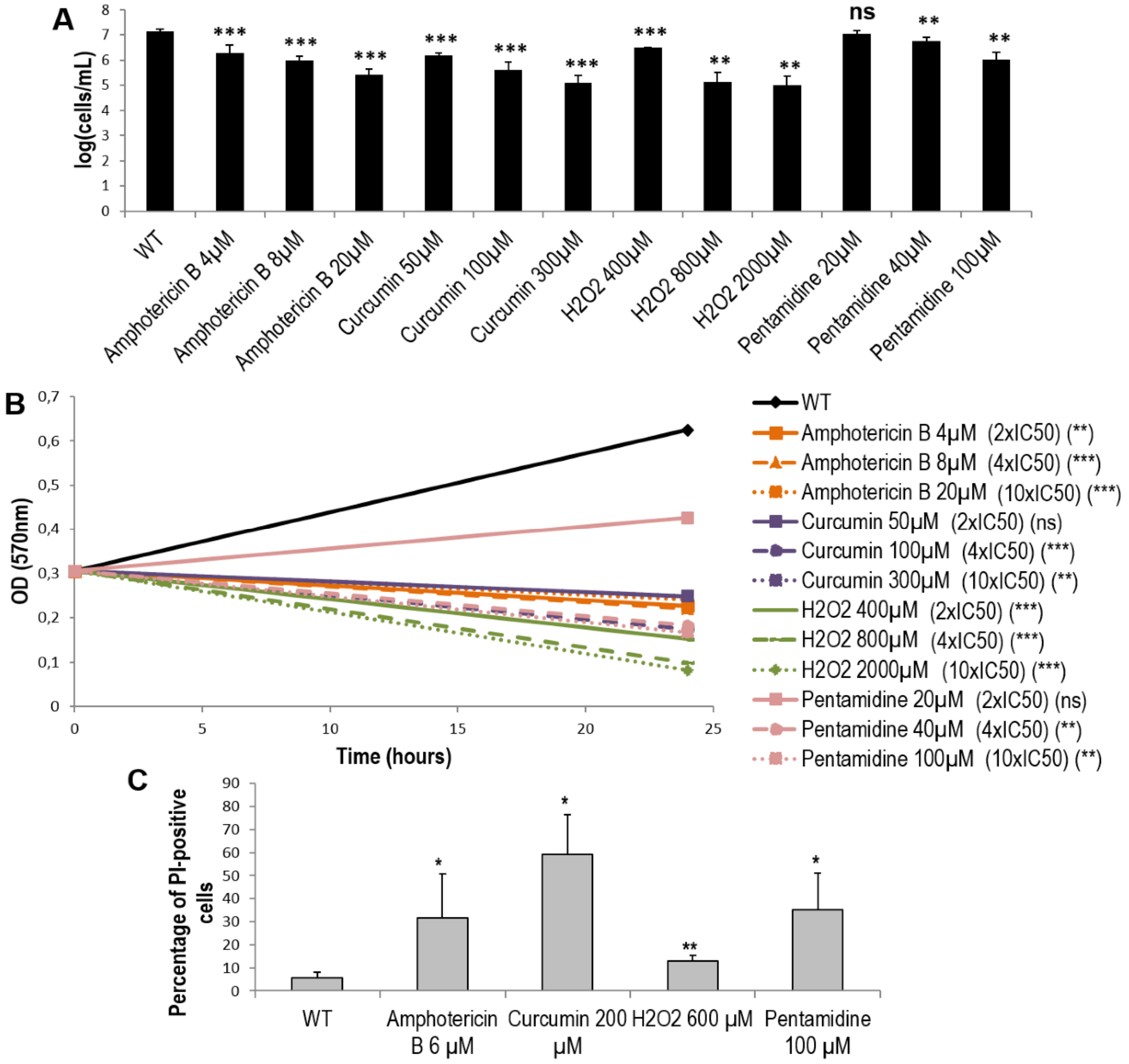

FIGURE S4: Amphotericin B, curcumin, H_2O_2 and pentamidine induce *Leishmania* cell death. (A) Cell concentration after culture for 24 h with amphotericin B, curcumin, H_2O_2 and pentamidine. All molecules induced cell growth inhibition at all concentrations tested, with the exception of pentamidine at 20 µM. Mean ± SD from a minimum of three independent experiments. Unpaired t-test between the condition of interest at 24 h and the control condition (WT) at 24 h. **(B)** Optical density, after the addition of MTT, of cells cultivated with various concentrations of amphotericin B, curcumin, H_2O_2 and pentamidine. All molecules were cytotoxic. A minimum of three independent experiments were carried out. Unpaired t-test between the condition of interest at 24 h and the same condition at 0 h. **(C)** Percentage of PI-positive cells after culture for 24 h with different molecules. The significant increase indicates loss of plasma membrane integrity, a feature of cell death. Unpaired t-test (compared to WT). ns not significant, * $p < 0.05$, ** $p < 0.01$, *** $p < 0.001$.

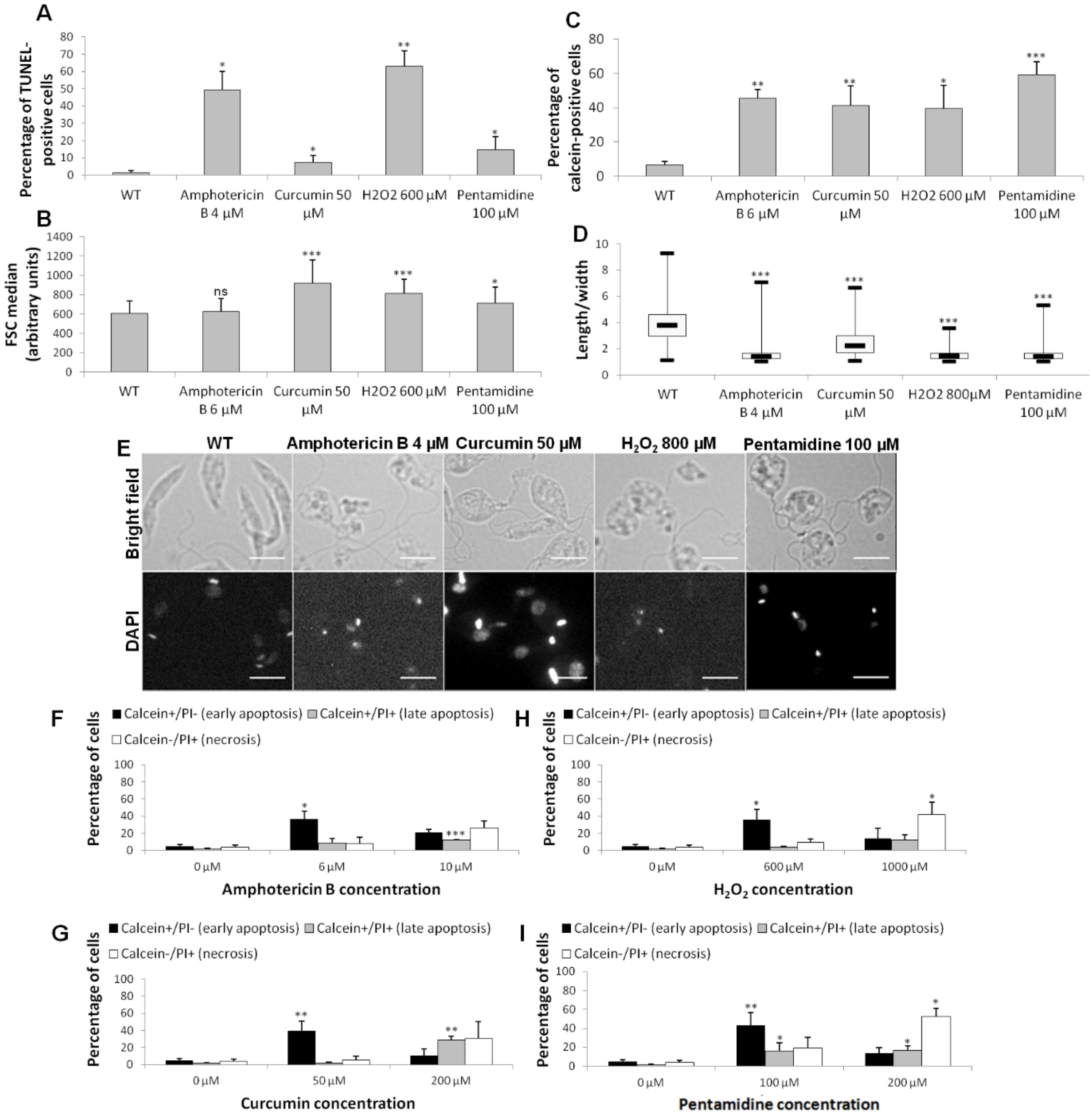

FIGURE S5: Amphotericin B, curcumin, H_2O_2 and pentamidine induce *Leishmania* apoptosis. (A) Percentage of TUNEL-positive cells after treatment for 24 h with amphotericin B, curcumin, H_2O_2 and pentamidine. The significant increase after the addition of the different molecules indicates DNA fragmentation. A minimum of 588 cells were counted from a minimum of three independent experiments. **(B)** FSC median assessed by flow cytometry of cells cultivated with various molecules. The increase in FSC median, observed with all molecules with the exception of amphotericin B at 6 µM, is indicative of cell shrinkage. Mean ± SD from a minimum of fourteen independent experiments. **(C)** Percentage of calcein-positive cells when culturing *L. major* cells with various molecules for 24 h (≥ three independent experiments). **(D)** Box plots representing length-to-width ratio of cells cultivated with different molecules. A significant rounding up was observed after the addition of all molecules for 24 h. A minimum of 527 cells were counted for each condition, from a minimum of three independent experiments. The thick line inside each box represents the median value; the lower and upper edge of each box indicates the 25th and 75th percentiles, respectively; the lower and upper whiskers (ends of the box arms) represent the minimum and maximum, respectively. **(E)** Microscopical observation of cells treated for 24 h with amphotericin B, curcumin, H_2O_2 or pentamidine and stained with DAPI, highlighting cell rounding up (bar = 5 µm). **(F-I)** Percentage of cells in early apoptosis (calcein+/PI-), in late apoptosis (calcein+/PI+) and in necrosis (calcein-/PI+) after treatment with various concentrations of amphotericin B **(F)**, curcumin **(G)**, H_2O_2 (H) or pentamidine **(I)**. A switch from early apoptosis to late apoptosis/necrosis was observed when the concentration of all molecules was increased. A minimum of two independent experiments was carried out for each concentration. Unpaired t-test; ns not significant, * $p < 0.05$, ** $p < 0.01$ and *** $p < 0.001$.
